# Resource allocations and disparities in the Brazilian health care system: insights from organ transplantation services

**DOI:** 10.1186/s12913-018-2851-1

**Published:** 2018-02-07

**Authors:** Eduardo J. Gómez, Sven Jungmann, Agnaldo Soares Lima

**Affiliations:** 1 Department of International Development, King’s College London, London, UK; 2 Lung Clinic Heckeshorn, HELIOS Kliniken GmbH, Berlin, Germany; 3 Department of Surgery, School of Medicine, Universidade Federal de Minas Gerais, Belo Horizonte, Brazil

**Keywords:** Brazil, Health care, Health policy, Health care financing, Organ transplantats, Health equity

## Abstract

**Background:**

To date, few studies have assessed how Brazil’s universal healthcare system’s (SUS, Sistema Único de Saúde) systemic, infrastructural, and geographical challenges affect individuals’ abilities to access organ transplantation services and receive quality treatment.

**Discussion:**

In this article we evaluated the existing literature to examine the impact that SUS has had on an increasingly important healthcare sector: organ transplantation services. We assess how equity challenges within the transplantation system can be explained by wider problems within SUS. Findings suggest stark disparities in access to transplantation services both within and across Brazil’s regions. We found that these regional differences are partially due to logistical challenges, especially in loosely populated areas but are also a consequence of disparities in resource allocations within SUS and under-capacitated health care facilities affecting transplantation services.

**Summary:**

We suggest that Brazil needs to improve its health outcome measurement system for organ transplantations and epidemiological surveillance, to gain more comprehensive and comparable data. Finally, we recommend policy strategies to reduce barriers to access to transplantation services by increasing transplantation service coverage in some areas and investing in emerging technologies.

## Background

Addressing healthcare disparities in emerging nations is a new area of scholarly research. Among the BRICS nations, Brazil has stood out as a government that, while in principle is committed to establishing an effective universal health care system, in practice has not been fully dedicated to achieving this objective. Inadequate funding, poor health care infrastructure, insufficient human resources, and geographic distance have negatively affected patient access to adequate health care services through the government’s decentralized universal health care system, namely SUS (*Sistema Único de Saúde*). To date, however, few have analysed the impact that SUS has had on an increasingly important healthcare sector: organ transplantation services. As the number of non-communicable diseases (NCDs) with potentially damaging effects on organs increases, such as diabetes, hypertension, and cancer, demands for transplantation services will inevitably rise. Yet, studies have paid insufficient attention to how SUS’ systemic, infrastructural, and geographical challenges affect individuals’ abilities to access transplantation services and receive quality treatment. We aim to fill in this lacuna in the literature by illustrating how regional differences in SUS human resources, medical infrastructure, waiting periods for medical attention and neurological exams lead to disparities in transplantation processes and provisions between the states. More specifically, Brazil’s wealthier southern states have seen better resources and infrastructure, in turn facilitating access and treatment in transplantation services; the converse holds for most poorer north-eastern states.^1^ We also desire to highlight that geographical factors matter: that is, patients in poorer rural areas confront transportation difficulties in obtaining access to transplantation services, which is less the case in urban areas. Scope: We focus on the Brazilian public healthcare system in its wider national context (e.g. overall infrastructure).

When conducting research for this article, the authors obtained referenced articles from personal databases of journal articles and reports, found mainly from on-line library systems and search engines, such as *PubMed* and *Google*. The creation of this personalized database occurred between 2013 and 2014. We used the keywords “Brazil” or “Brazilian” in combination with “Health Care”, “SUS”, and “Sistema Unico de Saude”, and “Transplantation”, “Solid Organ Transplant” (24, 2, and 15, respectively) in title, abstract, and body. Purely clinical publications that need to relate to system-level issues were excluded. *Google* was primarily used for targeted search queries such as “Brazilian Infrastructure.” Additionally, we also relied on our personal library on the topic and accessed databases such as the World Economic Forum Global Competitiveness Report, OECD StatExtracts, Instituto Brasileiro de Geografia e Estatística, Ministério da Saúde, Scientific Registry of Transplant Recipients to obtain specific statistics related to the issue.

The case of Brazil was chosen because of the authors’ experience conducting research in Brazil and our ability to easily obtain data. Brazil was also selected because of the absence of intra-regional comparisons in organ transplantation services and the need to further analyse how SUS has responded to increased demands for organ transplants. When analysing regional disparities in the provision and processes of transplantation services, we also used data from the *Associação Brasileira de Transplante de Órgãos* (ABTO) [1] in order to conduct a comparative quantitative assessment of these regional differences. Our factual statements were supported from qualitative evidence obtained from primary- and secondary sources, such as government documents and peer-reviewed journal articles. Finally, because much of our data was available on-line and/or from fieldwork, we did not find it necessary to conduct interviews with SUS workers and patients. Ethical considerations: This publication does not report on or involve the use of any animal or human data or tissue; instead, it relied solely on an analysis of existing records and published literature. Hence, no ethical approval was necessary.

## Explaining Brazil’s inequalities in organ transplant services

### Organ transplantations and health care inequalities

Building on Lancet and World Bank studies analysing the success and challenges of Brazil’s SUS [2–4] as well as other studies emphasizing the importance of situating particular health sectors within their broader health systems context [5–9], our analytical approach seeks to address how SUS’ systemic challenges have affected transplant services. We further contribute to this literature by illustrating how geographic (urban versus rural) differences in potential per capita organ donors and the availability of medical personnel accounts for regional disparities in providing transplantation services. Our approach therefore goes beyond mere descriptions of Brazil’s transplantation policies, as outlined by ABTO’s report *“Dimensionamento dos Transplantes”* [1].

Other scholars claim that Brazil succeeded in creating a well organized national transplantation system, making Brazil the second largest country in absolute numbers of kidney transplants in 2009 [7]. However, they also mention regional disparities in transplantation services consequent to the development status and “related mainly to differences in demographic density, GDP, and level of development” (p. 1370). Yet, these authors did not conduct a more rigorous analysis of how SUS’ systemic challenges, as well as geographic differences in transplantation infrastructure, accounts for these regional disparities; our article addresses these issues.

Finally, others claim that while the public sector is persistently underfinanced, there is an increasing subsidization of the private sector by the government [2]. In Brazil, for instance, private hospitals are often employed by SUS to perform liver transplants. This results in a further widening of the quality and accessibility gap between the public and private sector [10, 11]. Noronha and colleagues [10] note that of Brazil’s 6384 hospitals in 2010, 69% were private, with 31% of beds in the private sector being available to SUS through contracts. In December 2013, just 38% of beds were located in the public sector [12]. These issues, when combined with concerns of insufficient quality enforcements that lead to adverse health outcomes [13, 14], can partially explain seemingly rising levels of public dissatisfaction with the health system [3, 15].

### Systemic challenges for transplantations

In Brazil, the financing of SUS is mainly provided through federal, state, and municipal taxation as well as grants from the Ministry of Health (MOH) [16]. Since 2002 and Constitutional Amendment 29, the municipalities have been responsible for health care financing, allocating approximately 15% of their total tax revenue for SUS [16].

With respect to health policy management, the MOH, state, and municipal governments are all responsible, though in varying degrees. The MOH is mainly tasked with the responsibility of establishing technical policy norms and regulations; the state governments are responsible for technical assistance and consultation; while the municipal health departments manage and regulate policy implementation in SUS-run hospitals [17]. Municipal health departments also work with hospitals to provide primary care services, surgeries, prevention, and drug treatment [18]. The provision of transplantation services is financed by the MOH though managed by municipal health departments, in conjunction with SUS hospitals.

Transplantable organs are scarce and at any one time there are more patients waiting for a transplants than there are organs. To address this problem, transplantation programs must ensure that there is considerable public awareness around organ transplantation to ensure a constant flow of organs. Identifying suitable candidates for an organ is resource intensive. Extensive medical work-up is required to ensure that available organs are only transplanted in suitable recipients. Once a potential donor is identified, medical and surgical teams have to work quickly to extract usable organs and keep the *cold ischemic time* (period between organ extraction and transplant) as minimal as possible to increase the likelihood of successful transplantation. Patients on waiting lists need to be assessed carefully by specialists on a regular schedule and are required to be available on short notice, should an organ become available. After the transplant, regular follow-up assessments by expert clinicians are necessary to assess the organ’s functioning and viability.

An on-going challenge for the provision of transplantation services, however, has been the availability of adequate human resource personnel. In addition to a shortage of doctors and nurses [19], there is a dearth of neurologists and neurosurgeons, specialists that are needed to determine if a patient has clinically died, which requires an analysis of their cognitive brain capabilities, and if a transplant procedure can take place. As of 2011, the poorer northeast regions of Brazil had a low number of neurologists, on average between 11 and 50 for several northern states, with some poorer northern states, such as Roraima and Amapá having 1 to 10 neurologists [20]. Approximately 56.2% of neurologists reside in the more economically affluent southeast corridor of the nation, comprising the states of Rio Grande do Sul, São Paulo, Paraná, Santa Catarina, and Minas Gerais [20]; the north, conversely, only has approximately 1% [20].

In addition to a lack of adequate laboratories, blood circulation support networks, and intensive care units, there is an on-going shortage of essential health care infrastructure, such as beds and x-ray machines [19]. Again, the more affluent southern region fairs better in possessing these infrastructural resources when compared to the north [19].

Further problematic is the unequal distribution of national transplantation centres, known as the *Centrais de Notificação, Captação e Distribuição de Órgãos* (CNCDOs; Centres for Notification, Captivation and Distribution of Organs). The CNDO is mainly responsible for identifying potential organ donors, following brain death protocol, approaching the donor’s family to ask for an organ donation, blood examinations of organs received, computer storage of data, receipt and registration of organs, information that is used to determine those individuals eligible for transplants [21]. In addition to administrative personnel, CNDOs are often staffed with doctors, psychologists, and social workers. Once again, the CNDOs are mainly located in the more affluent north-eastern and southern regions: there are 19 CNDOs in this area versus 3 in the poorer northern states of Cuiabá, Manaus, and Belém [21]. These differences in accessing CNDOs contribute to growing disparities in the ability to donate and transplant organs. Furthermore, with the exception of Porto Alegre, São Paulo, and Rio de Janeiro, many CNDOs are located in hard to reach areas.

### Disparities in the demand and provision of organ transplantations

From an organizational perspective, Brazil’s states are grouped into five administrative regions [22]^2^:

1. *Central-West* (Distrito Federal, Goias, Mato Grosso, Mato Grosso do Sul),
2. *North-East* (Alagoas, Bahia, Ceará, Maranhão, Paraíba, Pernambuco, Piauí, Rio Grande do Norte, Sergipe),
3. *North* (Acre, Amapá, Amazonas, Pará, Randônia, Roraima, Tocantins),
4. *South-East* (Espirito Santo, Minas Gerais, Rio de Janeiro, São Paulo)*,* and
5. *South* (Paraná, Rio Grande do Sul, Santa Catarina).

Stark geographical and social inequalities in morbidity and mortality rates exist within and between these regions [2]. No comprehensive data for mortality and morbidity outcomes are available for Brazil, but the ABTO [1] provides data that yields insights on how these regional differences reflect in the transplantation system.

Firstly, the organ supply expresses strong regional heterogeneity. As Table 1 illustrates, the southern regions are generally able to provide more organs to those in need, particularly for kidneys and livers: while the demand for kidneys was met for 62% and 75% of patients registered in southern regions, only 13% to 27% of those living in the rest of Brazil received transplantation services in 2012 [1]. Not a single liver or heart transplant was performed in the entire northeast in 2012. In fact, several states did not provide any transplants for one or more types of organs (northern region: 7/7; north-eastern region: 7/9; central-western region: 3/4; south: 1/3; south-east 0/4 [1]).

**Table 1:** Mean number of estimated organ demand and supply across all states of each region in 2012 [1]

		Central-West	Northeast	North	Southeast	South
Cornea	Demand	316 (220-540)	204 (41-682)	531 (186-1262)	821 (562-962)	1808 (316-3714)
	Supply	425 (197-982)	69 (0-284)	343 (54-1084)	789 (475-1007)	1883 (269-5570)
	Ratio	1.34	0.34	0.65	0.96	1.04
Kidney	Demand	211 (147-360)	136 (27-455)	354 (124-841)	548 (375-642)	1206 (211-2476)
	Supply	52 (0-100)	18 (0-70)	94 (2-285)	409 (245-548)	744 (99-1947)
	Ratio	0.25	0.13	0.27	0.75	0.62
Liver	Demand	88 (61-150)	57 (11-350)	147 (52-350)	228 (156-267)	502 (88-1032)
	Supply	10 (0-39)	0 (0-0)	39 (0-160)	108 (106-112)	220 (32-586)
	Ratio	0.11	–	0.27	0.47	0.44
Heart	Demand	21 (15-36)	13 (3-45)	35 (12-84)	55 (37-64)	121 (21-248)
	Supply	5 (0-18)	0 (0-0)	5 (0-28)	12 (0-26)	32 (7-78)
	Ratio	0.24	–	0.14	0.22	0.26

Numbers in brackets express ranges. Numbers are rounded to whole numbers. *Ratios* were calculated by dividing supply by demand

For a transplant to take place, a clinician needs to quickly identify a deceased patient as a potential donor, and then seek consent from their family. Table 2 illustrates not only strong differences between notification rates within regions (the north and northeast fall behind the rest), but it also shows that the proportion of actual donations per notified potential donors to those in need is lower in northern regions [1].

**Table 2:** Number of notifications for potential donors, actual effective solid organ donations per 1000 inhabitants, and the proportion of actual donations per notifications in 2012 [1]

	Central-West	Northeast	North	Southeast	South
Notification	47.7	36.7	20.1	46.3	50
Actual donation	7.0	8.8	3.7	15.9	18.6
Donation/Notification	15%	24%	18%	34%	37%

Geographical and infrastructural differences account partially for these disparities. It is easier to provide transplantation services in more densely populated and better accessible areas. Patients need to travel less to attend their regular follow-up visits and there are more organ donors in close proximity, facilitating the deployment of surgical teams. Here, the southern and south-eastern regions of Brazil have a clear advantage. The *Instituto Brasileiro de Geografia e Estatística* [23] observed, for example, the highest density in the *south-eastern* region (87.0 inhabitants per km^2^), followed by the *southern* (48.6) and *north-eastern* (34.2) regions, with a steep decline in the *central-western* (8.8) and *eastern* regions (4.1). While the northern regions covers an area of nearly 3,9 M km^2^, the southeastern and southern regions only span over circa 0,9 M km^2^ and 0,6 M km^2^, respectively [23]. The south and southeast have much higher service coverage per 100,000 population than the rest. The southern region also has more follow-up care services per surface area than the southeast (although this does not necessarily imply an equal and equitable distribution).

Lima and colleagues [24] showed that in the south-east, rural dwellers in need for a liver transplant are often forced to migrate to cities in order to access health care services that are adequately equipped to provide for their treatment needs. These include regular follow-up consultations after the transplant. For many, however, migration is not an option: residents of poor municipalities are far less likely to be admitted to a tertiary clinic than residents of wealthier municipalities [2, 25], where transplantation services are more readily available.

Some of these challenges could be addressed by improving the transportation infrastructure, including public transport, which is one of Brazil’s major weaknesses: it ranks 114th out of 148 countries worldwide in the quality of overall infrastructure, 120th in road quality, and 123rd in air transport [26]. Those are crucial preconditions for rapid surgical team deployment and patient access to specialist services, which appear to be particularly problematic in the north: In the Amazonas, transplants are performed almost exclusively in the state capital. In Acre, surgical teams struggle to deploy to other hospitals for organ retrievals [1].

However, the north/south divide in access to transplantation services can not only be explained by logistical or geographic constraints. They also reflect different challenges in Brazil’s health care system.

Firstly, the low notification rates (clinicians reporting a potential donor) observed in many states (including the south [1]) might be consequential to reduced awareness of clinicians or insecurities regarding donation request practices. Private hospitals often appear to notify less than public hospitals [1].

More importantly, however, high family refusal rates seem to be a major issue. A lack of trust might be the key reason for this, either due to dissatisfaction with the perceived treatment quality prior to the brain death or due to negative press coverage over 20 years ago, which persists in the collective memory [1]. Although further research is needed, it appears that improvements of health care services unrelated to transplantations would yield positive externalities for the latter in terms of increased family consent to organ donations. Several scholars highlight the importance of public education and better counselling of families of potential donors [27–30] Brazil seems to require improvements in both areas [31].

Thirdly, delays in the diagnostic workup of potential donors form a major challenge in two states in the northern and one state in the north-eastern region. Acre (northeast), for example, relies on a laboratory in Goiânia for its histocompatibility analyses, while Paraíba (northwest) often lacks qualified personnel to diagnose brain death [1]. Delays in diagnostic workup can result in the loss of donors if blood circulation cannot be maintained long enough as is the case in one important emergency unit in Piauí lacks sufficient equipment to maintain blood circulation of deceased donors long enough for surgical teams to retrieve the organs [1].

This leads to the fourth problem: the lack equipment to carry out transplantations even reaches the level of profound structural problems in two states of the central-western and four in the north-eastern region, e.g., a lack of certified centres or dysfunctionalities along the entire transplantation system. However, “financing issues” were explicitly mentioned only for Pará [1]. None of these challenges were mentioned in the southern and south-eastern regions (although reporting bias is possible). Increased demand drives economies of scale through improved overall infrastructure and better supply of high-skilled surgical personnel. The previously presented challenges primarily create barriers that negatively affect the demand and supply of transplantation services.

However, financing disparities are another crucial factor: While public payments sufficiently cover the high cost of transplantation procedures [5], there are inequities favouring private health insurance holders said to have better access to preventive and treatment services, consequently demand health care services more often [2, 32], while receiving transplantations paid for by SUS [33]. The south-eastern region covers 11% of Brazil’s land surface – accounts for 43% of the population and 56% of GDP, while the north and north-east form the country’s poorest regions [2]. Since public spending for SUS accounts for only 3.1% of GDP [34] and around 59% of all spending on health care is private [35], this matters. It is more than in most other Latin American countries (e.g., Mexico with 47% [36]) or the United States (46% [34]).

This is not merely a matter of accessing inequalities, but Brazil also appears to have worse outcomes: its one-year survival rate is well below 80% [1], compared with around 85% in the USA [37]; although, making accurate comparisons is difficult.

## Conclusion

Current findings suggest stark disparities in access to transplantation services both within and across Brazil’s regions. These are partially due to logistical challenges, especially in loosely populated areas but are also a consequence of disparities in resource allocations within the health sector and under-capacitated basic health care facilities affecting transplantation services. Going forward, the MOH should aim at (a) narrowing the organ transplant supply gap, (b) improving health outcomes of transplants, and (c) decreasing barriers to access of transplantation services, particularly for the hardest to reach.

Brazil needs to improve its health outcome measurement system for organ transplantations, to gain more comprehensive and comparable data that also facilitates international comparisons. Currently, standard ratios are used to calculate organ needs estimate which do not reflect regional differences in the burden of disease. Improved epidemiological data collection and evaluation could help better inform health service planners. Comprehensive information on health care spending to the various sub-services should be made publicly available to allow for scholarly evaluations. This is particularly important as Brazil has a history of mismatching health care funding with the actual burden of disease [38].

Secondly, measures to increase public trust in health services might yield benefits to Brazil’s transplantation system. This can be improved by enhancing public awareness campaigns and intensifying donation request trainings already during undergraduate training.

Thirdly, greater efforts are possible to regionalize transplantation services, particularly regarding pre- and postoperative follow-up assessments. Here, Brazil has an opportunity to become a driver of innovation: for example, some departments are already spearheading telehealth projects to bring specialist health care to remote municipalities [39].

However, Brazil’s transplantation services can only improve within the tight constraints of the on-going weaknesses of SUS and transportation infrastructure. The geographic barriers to accessing transplantation services highlight the need to improve basic infrastructure (e.g. roads, public transport, and airports capable of nocturnal operation). Long waiting periods in hospitals, especially in the northeast, suggest a lack of adequate health care personnel and equipment (e.g. laboratories, life support machines for emergency departments); and a dearth of notification rates for the availability of organs also underscores the need to strengthen reporting systems for the availability of organs.

Finally, we hope that our analysis will inspire others to do the same. Given the burgeoning rise of non-communicable diseases in Brazil and other emerging economies, such as type-2 diabetes, heart disease, and cancer, the demand for organ transplants will increase. Escalating demands in transplantation services are indeed a by-product of the fast paced growth of NCDs. It would therefore behove researchers to analyse government commitment to strengthening transplantation services in other emerging economies experiencing a burgeoning growth of NCDs, such as China, India, and Mexico.^1^^,^^2^

## Abbreviations

ABTO: Associação Brasileira de Transplante de Órgãos (Brazilian Organ Transplantation Association)
BRICS: Acronym for an association of five major emerging national economies: Brazil, Russia, India, China and South Africa.
GDP: Gross Domestic Product
MOH: Ministry of Health
NCD: Non-communicable diseases
SUS: Sistema Único de Saúde (Brazilian Unified Health System)
USA: United States of America

## Footnotes

1 Of note is an important exception, Ceará, which is—together with Santa Catarina and Distrito Federal—among the national leaders in relative numbers of donations and performed transplants [1]. The reason for Ceará’s success is unknown, although evidence suggests that it is attributed to improved governance. For the purpose of this piece, the northeast will be regarded as one entity, since Ceará is still the exception from the rule.

2 NB: For the allocation of organs within transplants, Brazil is organized in four regional groups [40]. Allocation follows a tier system: if intrastate matching is impossible, regional, then national matching is attempted.
